# Patient characteristics, treatment patterns, and outcomes of Rickettsial diseases among a commercially insured population in the United States, 2005–2017

**DOI:** 10.1038/s41598-021-96463-9

**Published:** 2021-09-15

**Authors:** Alison M. Binder, Paige A. Armstrong

**Affiliations:** Rickettsial Zoonoses Branch, Division of Vector-Borne Diseases, Centers for Disease Control and Prevention, Atlanta, USA

**Keywords:** Infectious diseases, Bacterial infection, Epidemiology, Antimicrobial therapy

## Abstract

Rickettsial diseases (RDs) are transmitted to humans by ectoparasites, including ticks and fleas. Symptoms range from mild febrile illness, to severe disease or death. Doxycycline is the treatment of choice for patients of all ages; early treatment based on clinical diagnosis is critical to prevent severe outcomes. We conducted a descriptive analysis using insurance claims data captured by IBM MarketScan^®^ research databases to describe demographics, treatment patterns, and outcomes of patients diagnosed with RDs in the United States during 2005–2017. Overall, 14,830 patients had a RD diagnosis during 2005–2017; 7,517 (50.7%) spotted fever rickettsiosis (SFR), 4,571 ( 30.8%) ehrlichiosis, 1,362 (9.2%) typhus group rickettsiosis (TGR), and 1,193 (8.0%) other rickettsial diseases. Among all patients diagnosed, 53.1% received doxycycline. Prescription rates varied by diagnosis and age; 24.1% of TGR and 61.1% of SFR patients received doxycycline; 23.9% of persons < 8 years received doxycycline, compared with 47.7% for 8–17 years, and 55.4% for ≥ 18 years. RDs are frequently diagnosed in the outpatient population; however, providers prescribed the recommended treatment to about half of patients. Continued education of treatment recommendations is critical to prevent severe outcomes.

## Introduction

Rickettsial diseases are vector-borne diseases caused by various bacterial species from the genera *Rickettsia*, *Ehrlichia*, and *Anaplasma*^1^. The primary ectoparasites implicated in transmission are ticks, fleas, lice, and mites. In the United States, the nationally notifiable tickborne rickettsial diseases (TBRDs) are spotted fever rickettsioses (SFR; including Rocky Mountain spotted fever [RMSF, caused by infection with *R. rickettsii*]), ehrlichiosis (caused by various *Ehrlichia* species including *E. chaffeensis* and *E. ewingii*), and anaplasmosis (caused by *Anaplasma phagocytophilum*). During 2014–2017, TBRD case counts have increased over fivefold, from 2,588 to 13,652, with the largest proportion reported in the SFR category^1^. Non-notifiable rickettsial diseases, and those transmitted by ectoparasites other than ticks are reportable in some states, but without national data, estimates of burden are difficult to ascertain. Typhus group rickettsioses (TGR), including epidemic (louseborne) typhus (caused by *R. prowazekii* infection) and murine (fleaborne or endemic) typhus (caused by *R. typhi* infection), are less commonly diagnosed in the United States, but remain important causes of disease in certain regions^2^.

Rickettsial diseases often present as a nonspecific acute febrile illness 1–2 weeks after exposure. Clinical symptoms range from mild to severe, and often include headache, malaise, and arthralgia. Many rickettsial diseases are also accompanied by a rash, but characteristics and prevalence vary by condition^1^. In cases of RMSF, the distinguishing maculopapular to petechial rash typically appears on day 2–4 of illness^2^, but 10–20% of patients will not develop a rash^3,4^. Rash is less common in anaplasmosis and ehrlichiosis. The early nonspecific presentation of rickettsial diseases can cause delays in diagnosis and initiation of treatment with doxycycline, resulting in severe outcomes and death^5^. RMSF is the most fatal rickettsial illness, with an estimated case fatality rate in the U.S. of approximately 5–10%; rates reach 40–50% when treatment is not initiated^4^. Mortality rates are much lower for other rickettsial diseases including ehrlichiosis, anaplasmosis, and typhus, but complications, such as meningoencephalitis, acute respiratory distress syndrome, and renal failure occur when treatment is delayed^6^.

The Centers for Disease Control and Prevention (CDC) and the American Academy of Pediatrics (AAP) recommend doxycycline as first-line treatment for patients of all ages with suspected rickettsial disease^7,8^. Early treatment with doxycycline based on clinical diagnosis is important to prevent severe and fatal outcomes. Lack of treatment within five days of illness is the single most important predictor of a fatal outcome in cases of RMSF^5^. Historically, use of tetracycline antibiotics (including doxycycline) was not widely accepted in children < 8 years of age due to concerns of tooth staining and enamel hypoplasia^9^. However, studies have shown that the benefits of therapy, in the short course needed to treat rickettsial diseases, exceed risk^4,10–12^. Recent studies have disproven the association between doxycycline and tooth staining^13–15^.

Despite current recommendations and available information on the severity of rickettsial diseases, studies show providers delay antirickettsial treatment. A 2009 questionnaire conducted in Tennessee showed only 39% of participating providers reported they would prescribe doxycycline to children < 8 years of age in whom they suspected RMSF^2^. More recently, a 2012 national DocStyles survey of healthcare providers revealed only 35% of respondents knew doxycycline is the appropriate treatment for RMSF in children < 8. Pediatricians responded correctly more often compared to other providers (51% vs 32%, respectively)^9^. This persistent misunderstanding is particularly concerning since children < 10 years of age represent less than 6% of all RMSF cases, but 22% of deaths^13^.

While published studies have described provider awareness and treatment practices for RMSF, very limited studies have been conducted to describe treatment patterns and outcomes for other rickettsial diseases^6,16–18^; current studies also have limited time horizons, and the most recent U.S. survey was conducted in 2012. Additionally, while SFR, ehrlichiosis, and anaplasmosis are notifiable in the U.S., national surveillance platforms do not collect data on treatment patterns, and neither TGR, nor other rickettsiosis, such as rickettsialpox, are currently nationally notifiable. This study provides a comprehensive description of the demographics, treatment patterns, and outcomes of rickettsial diseases in the U. S. using data captured in a large administrative claims database, while also identifying potential gaps in provider knowledge to guide targeted clinical education efforts and improve patient outcomes.

## Methods

### Data source and patient identification

We conducted a retrospective, descriptive analysis of the IBM Watson Health MarketScan® Commercial Claims and Encounters database. The database captures employer-sponsored medical billing data from more than 300 employers and 15 health plans, and includes enrollment information from employees, retirees aged < 65 years, former employees, and their spouses and dependents from all states. Data contains inpatient and outpatient medical claims, diagnoses, diagnostic tests and procedures, prescriptions, and cost assessments. Data are statistically de-identified and compliant with HIPAA, and were acquired for this analysis through funding provided by the Centers for Disease Control and Prevention. Data were used under agreement for the current study, and so are not publicly available.

All patients with outpatient medical encounters associated with a rickettsial disease between January 1, 2005 and December 31, 2017, identified using the International Classification of Diseases, Ninth and Tenth Revisions, Clinical Modification (ICD-9/10-CM) codes, were eligible for inclusion (Supplemental Table 1). ICD-9/10-CM codes are included in inpatient and outpatient medical encounter records, with up to 15 unique codes in each inpatient record, and up to four in each outpatient record; prior to October 1st, 2015, ICD-9-CM codes were used, and after which ICD-10-CM were used^19^. The first claim associated with a rickettsial disease was considered the index diagnosis. To ensure we were accurately capturing a unique diagnosis, treatment, and outcome, we excluded patients with ≤ 12 months of continuous enrollment prior to their index diagnosis, and patients with ≤ 90 days of follow-up after their index date. We excluded any patients hospitalized with a rickettsial disease in the 12-month pre-index period as our goal was to assess treatment in those newly diagnosed with a rickettsial disease. Lastly, all patients were required to have outpatient pharmaceutical information available during the study period to accurately assess treatment patterns during the study period.

### Study variables and analysis

The following mutually exclusive diagnostic categories were created based on rickettsial disease specific ICD-9/10-CM codes associated with the index diagnosis: spotted fever rickettsioses (SFR), TGR, ehrlichiosis (includes anaplasmosis), and other rickettsial diseases (Supplemental Table 1). Demographic and geographic characteristics associated with the index diagnosis were summarized for all patients. Geographic data were summarized by U.S. Census region and state, when available.

The primary outcome was rate of doxycycline prescription with index rickettsial disease diagnosis. Prescriptions are not directly linked to healthcare encounters; to capture prescriptions potentially associated with the index diagnosis, we included prescriptions filled within 30 days of the visit, creating a 60-day window around the index diagnosis. This approach was similar to efforts using administrative claims data to identify and describe Lyme disease and typhus group rickettsiosis diagnoses^20–23^. We used National Drug Codes (NDCs) specific to doxycycline to identify all applicable prescriptions. Inpatient pharmaceutical claims are not captured in the database, and so were not evaluated in this study. We calculated time from index diagnosis to prescription claim for all patients, and by diagnostic and age groups (< 8 years, 8–17 years, ≥ 18 years). Age groups were chosen because doxycycline had previously been contraindicated in children < 8 years of age, and because diagnostic and prescribing practices differ between adult and pediatric patients. We also examined pharmaceutical claims for systemic antibiotics other than doxycycline prescribed in conjunction with a rickettsial diseases diagnosis.

We assessed the secondary outcomes of hospitalization and discharge status during the post-index period for all patients, and by diagnostic group. Patients requiring admission were included in this sub-analysis if the hospitalization was associated with any rickettsial disease ICD-9/10-CM code and occurred within 30 days following their index (outpatient) diagnosis.

Descriptive analyses were performed for all study variables, treatment patterns, and outcomes. All data management and analyses were conducted using SAS version 9.4 (SAS Institute, Cary, NC, USA).

### Ethical considerations and approval

This study was deemed research not involving human subjects under 45 CFR 46.102(f) according to the CDC Human Research Protection Office; approval from an institutional review board was not required.

### Disclaimer

The findings and conclusions of this report are those of the authors and do not necessarily represent the official position of CDC.

## Results

### Study population

**Figure 1:**
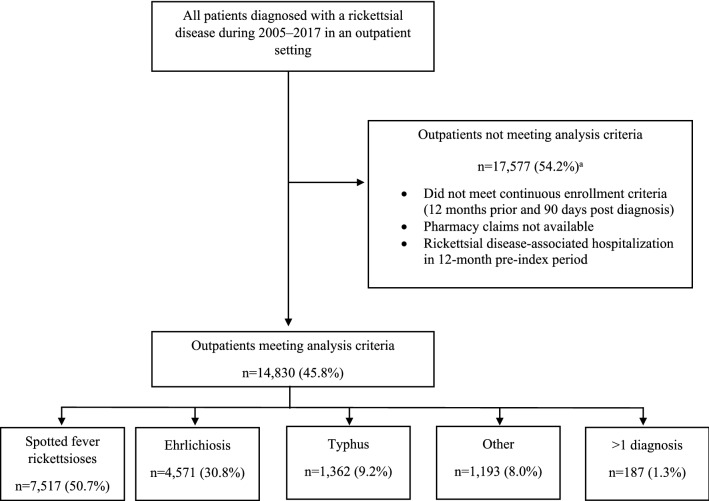
Selection criteria for outpatients diagnosed with rickettsial diseases from a large, commercially insured population—United States, 2005–2017. ^a^Exclusion criteria are not mutually exclusive; outpatients could be excluded for failing to meet more than one criteria listed.

In total, 14,830 patients with a rickettsial disease diagnosis met all selection criteria during 2005–2017. Most patients included were in the SFR diagnostic category (7,517; 50.7%), followed by ehrlichiosis (4,571; 30.8%), TGR (1,362; 9.2%), and other rickettsial diseases (1,193; 8%); patients with codes in more than one diagnostic category (187, 1.3%) were infrequent (Fig. 1).

### Cohort characteristics

The median age at diagnosis for all patients was 45 years (IQR: 29–55 years). Median age in years for SFR patients was 44 (IQR: 29–54); for ehrlichiosis patients, 47 (IQR: 32–56); for TGR patients, 42 (IQR: 20–53); and for other rickettsial diseases, 43 (IQR: 24–54). When stratifying by age group, TGR had the highest proportion (8.4%) of persons < 8 years of age, compared to SFR (4.7%), ehrlichiosis (3.0%), and other rickettsial diseases (7.4%). Male sex was more common among all patients (50.4%) and among SFR (53.6%), while female sex was predominant among ehrlichiosis (51.4%), TGR (57.1%), and other rickettsial disease diagnoses (54.1%). The majority (94.1%) of all patients were diagnosed in a physician’s office compared to the emergency department (Table 1).Geographically, the majority of TBRD diagnoses were associated with the South U.S. Census region (47.2%), followed by the Northeast (32.2%); the West region had the fewest diagnoses (5.4%; Table 1). Similarly, the majority of SFR (65.3%), TGR (48.2%), and other TBRD diagnoses (50.8%) were in the South; only ehrlichiosis (including anaplasmosis) showed a different distribution, with 65.0% of diagnoses associated with the Northeast region.

**Table 1:** Characteristics of outpatients diagnosed with rickettsial diseases in a large, commercially insured population—United States, 2005–2017.

	All outpatients	Spotted fever rickettsioses	Ehrlichiosis (including anaplasmosis)	Typhus	Other diagnosis	> 1 diagnosis
	(n = 14,830)	(n = 7,517)	(n = 4,571)	(n = 1,362)	(n = 1,193)	(n = 187)
Age (years) at index, median (IQR)	45 (29–55)	44 (29–54)	47 (32–56)	42 (20–53)	43 (24–54)	43 (33–53)
**Age group**						
< 8 years	699 (4.7)	353 (4.7)	138 (3.0)	115 (8.4)	88 (7.4)	5 (2.7)
8–17 years	1,475 (9.9)	731 (9.7)	427 (9.3)	190 (14.0)	118 (9.9)	9 (4.8)
≥ 18 years	12,656 (85.3)	6,433 (85.6)	4,006 (87.6)	1,057 (77.6)	987 (82.7)	173 (92.5)
**Male sex**	7,475 (50.4)	4,027 (53.6)	2,220 (48.6)	584 (42.9)	548 (45.9)	96 (51.3)
**Geographic region**						
Northeast	4,775 (32.2)	1,127 (15.0)	2,971 (65.0)	346 (25.4)	276 (23.1)	55 (29.4)
Midwest	1,915 (12.9)	890 (11.8)	679 (14.9)	158 (11.6)	160 (13.4)	28 (15.0)
South	6,995 (47.2)	4,909 (65.3)	729 (15.9)	657 (48.2)	606 (50.8)	94 (50.3)
West	800 (5.4)	358 (4.8)	126 (2.8)	188 (13.8)	119 (10.0)	9 (4.8)
**Setting of diagnosis**						
Emergency room	877 (5.9)	370 (4.9)	401 (8.8)	50 (3.7)	53 (4.4)	3 (1.6)
Outpatient office	13,953 (94.1)	7,147 (95.1)	4,170 (91.2)	1,312 (96.3)	1,140 (95.6)	184 (98.4)

Data are presented as No. (%) unless otherwise indicated.

### Treatment and outcomes

**Figure 2:**
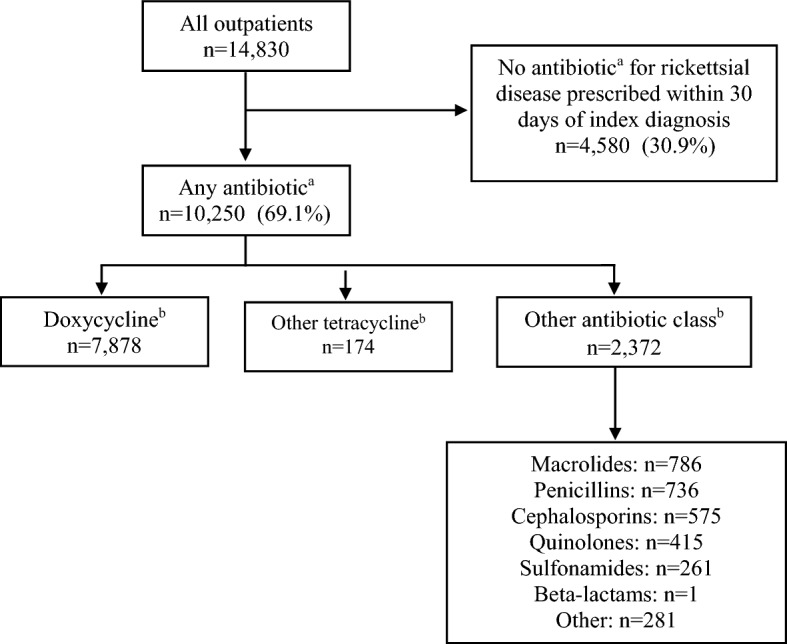
Outpatients diagnosed with a rickettsial disease treated with antibiotics within 30 days of index rickettsial disease diagnosis date in a large, commercially insured population — United States, 2005–2017. ^a^Prescription claims made from 30 days before to 30 days following index rickettsial disease diagnosis date. ^b^Antibiotic subgroups are not mutually exclusive; patients can be counted in more than one group.

Most patients (10,250, 69.1%) had a prescription for a systemic antibiotic within 30 days of their index rickettsial diagnosis; of these, 7,878 (76.9%) were prescribed doxycycline (Fig. 2). A small portion were prescribed a different tetracycline (174; 1.7%), and 2,372 (23.1%) were prescribed an antibiotic other than a tetracycline. The most common prescriptions among other antibiotic classes were macrolides (786, 33.1%), penicillins (736, 31%), cephalosporins (575, 24.2%), quinolones (415, 17.5%), and sulfonamides (261; 11%).

Considering only the recommended treatment for rickettsial disease, 53.1% of all patients (N = 7,878) diagnosed with a rickettsial disease received doxycycline (Table 2). Among patients receiving doxycycline, 82.8% received it within 14 days of the index diagnosis; 68.9% were within 7 days. Rates of prescription varied by diagnostic group; the lowest was 24.1% for TGR patients, 34.8% for other rickettsial diseases, 54.2% for ehrlichiosis, and the highest was 61.1% for SFR. Rates also varied considerably across age groups, with only 23.9% of persons < 8 years receiving doxycycline (94.0% of those received it within 14 days of index date), compared to 47.7% among 8–17 years (89.1% within 14 days of index date), and 55.4% of those ≥ 18 years (81.9% within 14 days of index date). The median days’ supply was 10 days for < 8 years and 8–17 years, but longer (14 days) for ≥ 18 years of age. Supplemental Fig. 1 shows the distribution of intervals from index diagnosis date to doxycycline prescription by age group.

**Table 2:** Doxycycline treatment patterns by diagnostic and age groups among outpatients diagnosed with rickettsial diseases in a large, commercially insured population — United States, 2005–2017.

**Doxycycline prescription rates**	
**Outpatients with doxycycline by diagnostic group**^**a**^	
**All**	7,878 (53.1)
Spotted fever rickettsioses (n = 7,517)	4,595 (61.1)
Ehrlichiosis (n = 4,571)	2,476 (54.2)
Typhus (n = 1,362)	328 (24.1)
Other rickettsial diseases (n = 1,193)	415 (34.8)
> 1 diagnostic group (n = 187)	64 (34.2)
**Outpatients with doxycycline by age group**^**a**^	
< 8 yrs. (n = 699)	167 (23.9)
8–17 yrs. (n = 1,475)	704 (47.7)
≥18 yrs. (n = 12,656)	7,007 (55.4)
**Time from index date to prescription (days)**	
Median (IQR)	0 (-9–0)
n, % of patients with prescription within ± 14 days of index date	6,520 (82.8)
n, % of patients with prescription within ± 7 days of index date	5,427 (68.9)
**Characteristics of first doxycycline prescription by age group**	
** < 8 yrs. (n = 167)**	
**Time from index date to prescription (days)**	
Median (IQR)	0 (-1–0)
n, % of patients with prescription within ± 14 days of index date	157 (94.0)
Days’ supply, median days (IQR)	10 (7–10)
ROA	
IV	0 (0)
Oral	167 (100)
Dosing and treatment patterns	
100 mg PO 10 days	6 (3.6)
Suspension PO 10 days	65 (38.9)
**8–17 yrs. (n = 704)**	
**Time from index date to prescription (days)**	
Median (IQR)	0 (-3–0)
n, % of patients with prescription within ± 14 days of index date	627 (89.1)
Days’ supply, median days (IQR)	10 (10–14)
ROA	
IV	1 (0.1)
Oral	703 (99.9)
Dosing and treatment patterns	
100 mg PO 10 days	442 (62.3)
**≥18 yrs. (n = 7,007)**	
Time from index date to prescription (days)	
Median (IQR)	-1 (-10–0)
n, % of patients with prescription within ± 14 days of index date	5,736 (81.9)
Days’ supply, median days (IQR)	14 (10–15)
ROA	
IV	3 (0.1)
Oral	7,004 (99.9)
Dosing and treatment patterns	
100 mg PO 10 days	6,028 (86.0)

Data are presented as No. (%) unless otherwise indicated.

*ROA* route of administration, *IV* intravenous, *mg* milligrams, *PO* by mouth.

Recommended dosing is 100 mg twice daily for adults or children ≥ 45 kg, and 2.2 mg/kg/dose twice daily for children < 45 kg. Duration of treatment is 5–7 days or at least 3 days after fever subsides or patient shows signs of clinical recovery.

^**a**^Percentages reflect the proportion of patients within that diagnostic or age category who received the recommended dosing and duration of doxycycline. Remaining patients either received a non-recommended dosing/duration of doxycycline or no prescription was identified in the database.

We identified 169 rickettsial disease-related hospitalizations within 30 days following the outpatient index diagnosis. Of these, SFR patients were the most frequently hospitalized (69, 40.8%), followed by ehrlichiosis (63, 37.3%), TGR (19, 11.2%), and other rickettsial diseases (14, 8.3%) (Table 3). The median time to hospitalization following index date was 1 day (IQR 1–2 days), and the median length of stay was 2 days (IQR 1–4 days). Most hospitalized patients (144, 85.2%) had evidence of doxycycline prescription within 30 days of their index date. Among hospitalized patients with known discharge status (156, 92.3%), 142 (91.0%) were discharged to home or self-care. No records of in-hospital death were identified (Table 3).

**Table 3:** Characteristics of outpatients with a rickettsial disease-associated hospitalization within 30 days following index date in a large, commercially insured population — United States, 2005–2017.

	**Patients hospitalized** **(n = 169)**
**Diagnostic group**	
Spotted fever rickettsioses	69 (40.8)
Ehrlichiosis	63 (37.3)
Typhus group rickettsioses	19 (11.2)
Other rickettsial diseases	14 (8.3)
> 1 diagnostic group	4 (2.4)
**Age group**	
< 8 years	5 (3.0)
8–17 years	15 (8.9)
≥ 18 years	149 (88.2)
**Characteristics of all hospitalized patients**	
Doxycycline prescribed	114 (85.2)
Time from outpatient diagnosis to hospitalization, days	
Median	1
Range	1–24
IQR	1–2
Length of stay, days	
Median	2
Range	1–23
IQR	1–4
Discharge status (n = 156)	
Discharged to home or self-care	142 (91.0)
Other discharge	14 (8.3)
In-hospital death	0 (0)

Data are presented as No. (%) unless otherwise indicated.

### Annual trends

**Figure 3:**
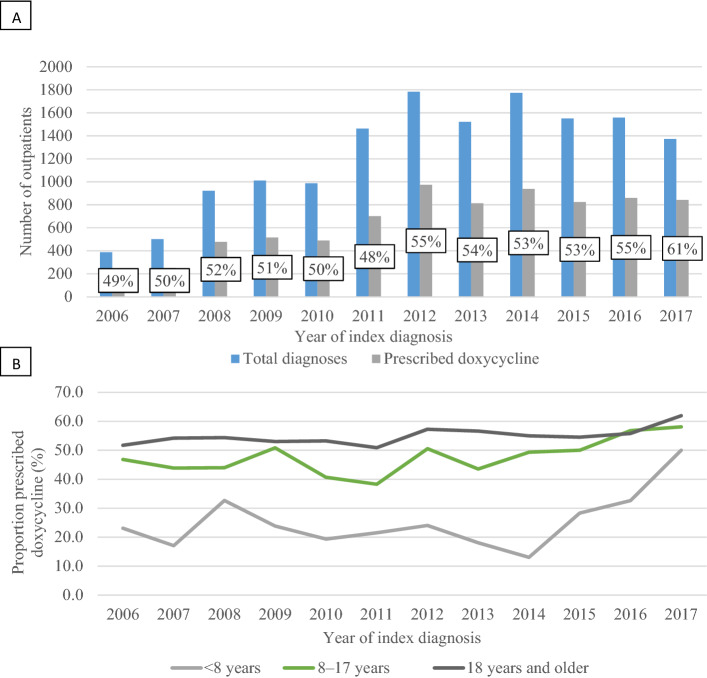
Number of outpatients with rickettsial diagnosis and percentage receiving doxycycline in a in a large, commercially insured population—United States, 2005–2017; by year (**A**) and by age group (**B**). Data shown does not include 2005 since patients were required to have one-year continuous enrollment in the databases prior to their diagnosis index date; therefore the earliest possible diagnosis is 1/1/2006.

The number of incident rickettsial diagnoses increased during the study period, from 388 in 2006 to a peak of 1,774 in 2014, to 1,372 in 2017. The mean percentage of persons with an index diagnosis of rickettsial disease receiving doxycycline was 52.5% annually; ranging from 49.2% in 2006 to 61.4% in 2017 (Fig. 3a). The prescription rate during 2006–2017 varied by age, with children < 8 years demonstrating the largest percent increase (from 23.1 to 50) compared to children 8–17 years (46.8 to 58.1), and adults ≥ 18 years (51.7 to 61.9) (Fig. 3b).

## Discussion

Early symptoms of rickettsial diseases are often difficult to distinguish from other etiologies of febrile illness. However, they remain a treatable cause of disease, and early doxycycline administration is key. Furthermore, only a portion of rickettsial diseases are nationally notifiable, leaving a gap in our understanding of national trends among categories such as TGR. Even then, national surveillance data does not provide comprehensive understanding of treatment patterns. Our results indicate rickettsial diseases continue to be an important cause of illness in the U. S., and targeted education of healthcare providers to reinforce treatment guidelines is needed to increase appropriate management.

Doxycycline is the recommended treatment of choice in persons of all ages in whom a rickettsial disease is suspected. And yet, only half of the patients diagnosed with a rickettsial disease were prescribed the indicated treatment. Even when an antibiotic was prescribed, nearly one quarter received an antibiotic with either no antirickettsial activity, or in the case of sulfonamides, that could be harmful as sulfonamides can cause more several illness or death for patients with ehrlichiosis or RMSF^7,24–29^. There are no alternative treatments as effective as doxycycline at preventing severe outcomes and death, and use of alternative antibiotics suggests a lack of familiarity with treatment recommendations^7^. There is also a clear disparity between the proportion of children < 8 years of age (24%) receiving doxycycline compared with patients ≥ 18 (55%), possibly due to outdated perceptions of risk to teeth and bone development. More reassuring is the increasing rate of treatment with doxycycline in the < 8 year age group, especially in recent years, suggesting efforts to disseminate findings of studies refuting detrimental effects to teeth may be permeating practice patterns^9^. However, the absolute numbers are still low. The recommendation to treat patients of all age with suspected rickettsial diseases with doxycycline is a key clinical point that should be incorporated into healthcare provider education.

Trends over time demonstrate that largely practice is not changing and nearly half of providers are not adhering to the recommendation that treatment should be based on clinical diagnosis, as currently available diagnostics often do not return actionable results in the time period during which treatment would be most effective. Especially pertinent is the case of RMSF, where initiation of treatment beyond day 5 of illness is the most important risk factor for fatal outcome^4^. It is possible providers are assigning a diagnostic code while awaiting confirmatory results. This may represent a lower level of clinical suspicion or unfamiliarity with rickettsial diagnostics and the need for early treatment.

Since we lack national-level data on TGR, this provides the largest current summary of national trends and treatment for the disease group. TGR had the largest proportion of patients < 8 and 8–17 years of age of all the rickettsial disease; however, children still represent a minority of those diagnosed (8.4% and 14%, respectively). Most concerning was that patients with a TGR diagnosis were least likely of all rickettsial diseases to receive doxycycline with only 24.1% treated. The fact that less than one quarter of the patient diagnosed with a treatable disease are receiving the recommended antibiotic suggests a potential gap in provider knowledge.

While administrative claims data are typically robust and can provide insights into provider diagnostic and treatment patterns, its use for epidemiologic research is subject to limitations. First, the data used for this study represents a convenience sample of persons < 65 years of age with commercial health insurance. Medicaid or Medicare enrollees, military personnel, or those without insurance were not included; risk for rickettsial disease might differ among these populations. Therefore, the results presented might not be generalized to the overall U.S. population.

Administrative claims data are generated for reimbursement using diagnostic codes, which are provided by health care providers or billing specialists to insurance companies and are thus subject misclassification, and diagnostic data is not available to confirm. The use of a single diagnostic code in this study could lead to inclusion of a higher number of false-positives since diagnosis at the time of visit would most likely be on the basis of clinical criteria with confirmatory laboratory diagnosis available days to weeks later. A confirmatory laboratory diagnosis is most commonly provided when a convalescent specimen is tested and compared to an acute specimen taken 4–6 weeks prior to quantify the patient’s antibody response, with a fourfold rise in immunoglobulin-G antibodies specific to rickettsial pathogens confirming exposure^7^. National surveillance data for SFR has shown that the majority of cases reported in the United States have only one serologic test completed, and thus do not meet confirmatory criteria. However, approximately 25% of outpatients included in this analysis had a second TBRD diagnostic code within one year of their index date^30,31^. Despite these challenges in diagnostic confirmation, the decision to treat for a TBRD should be primarily based on clinical criteria, with the recommendation to initiate doxycycline while confirmatory lab results are pending. It is possible a non-specific ICD-9/10 code was assigned to an initial visit, and later TBRD confirmed by diagnosis; to address this, we used a 30-day window surrounding the index date to ensure capture of treatment that may have occurred after laboratory results. The majority of patients treated with doxycycline demonstrated treatment was received within 14 days of the index date; this information, coupled with the retrospective aspect of TBRD confirmatory diagnoses, decreases the possibility of misclassification of patients included in this analysis.

## Conclusion

Our study provides national-level data not available through standard surveillance, as well as a comprehensive look at treatment practices across all rickettsial diseases. Trends in both treatment and diagnosis suggest a need for increased provider awareness of clinical presentation, utility of diagnostics, and treatment guidelines. Opportunities for continuing education are available, and provide key information on rickettsial diseases, such as RMSF (www.cdc.gov/rmsf/resources/toolkit.html; www.cdc.gov/rmsf/resources/module.html). However, additional effort to increase presence in curriculum, and local and state-level outreach will also help ensure adherence to guidelines, and reduction in morbidity and mortality from treatable rickettsial diseases.

## Supplementary Information

Supplementary Information 1.

Supplementary Information 2.
