# Supplementary material for: Patient characteristics, treatment patterns, and outcomes of Rickettsial diseases among a commercially insured population in the United States, 2005–2017
Source: Sci Rep. 2021 Sep 15;11:18382. doi: 10.1038/s41598-021-96463-9 (PMC8443668; doi:10.1038/s41598-021-96463-9)
Supplement: Supplementary file 1 — Supplementary Information 1. [file 41598_2021_96463_MOESM1_ESM.docx]

**Supplementary table 1: ICD-9/10-CM codes associated with rickettsial diseases and construction of mutually exclusive diagnostic categories for analysis**

|  | **Code** | **Description** | **Diagnostic category** |
| --- | --- | --- | --- |
| **ICD-9-CM** | 080 | Louse-borne (epidemic) typhus | Typhus |
|  | 081 | Other typhus | Typhus |
|  | 081.0 | Murine (endemic) typhus | Typhus |
|  | 081.1 | Brill’s disease | Typhus |
|  | 081.2 | Scrub typhus | Typhus |
|  | 081.9 | Typhus, unspecified | Typhus |
|  | 082 | Tick-borne rickettsioses | Spotted fever rickettsioses |
|  | 082.0 | Spotted fevers | Spotted fever rickettsioses |
|  | 082.1 | Boutonneuse fever | Spotted fever rickettsioses |
|  | 082.2 | North Asian tick fever | Spotted fever rickettsioses |
|  | 082.3 | Queensland tick typhus | Spotted fever rickettsioses |
|  | 082.8 | Other specified tick-borne rickettsioses | Spotted fever rickettsioses |
|  | 082.9 | Tick-borne rickettsiosis, unspecified | Spotted fever rickettsioses |
|  | 082.4 | Ehrlichiosis | Ehrlichiosis |
|  | 082.40 | Ehrlichiosis, unspecified | Ehrlichiosis |
|  | 082.41 | Ehrlichiosis, *E. chaffeensis* | Ehrlichiosis |
|  | 082.49 | Other ehrlichiosis | Ehrlichiosis |
|  | 083 | Other rickettsioses | Other rickettsial disease |
|  | 083.1 | Trench fever | Other rickettsial disease |
|  | 083.2 | Rickettsialpox | Other rickettsial disease |
|  | 083.8 | Other specified rickettsioses | Other rickettsial disease |
|  | 083.9 | Rickettsiosis, unspecified | Other rickettsial disease |
| **ICD-10-CM** | A75 | Typhus fever | Typhus |
|  | A75.0 | Epidemic louse-borne typhus fever due *to Rickettsia prowazekii* | Typhus |
|  | A75.1 | Recrudescent typhus [Brill's disease] | Typhus |
|  | A75.2 | Typhus fever due to *Rickettsia typhi* | Typhus |
|  | A75.3 | Typhus fever due to *Rickettsia tsutsugamushi* | Typhus |
|  | A75.9 | Typhus fever, unspecified | Typhus |
|  | A77 | Spotted fever (tick-borne rickettsioses) | Spotted fever rickettsioses |
|  | A77.0 | Spotted fever due to *Rickettsia rickettsii* | Spotted fever rickettsioses |
|  | A77.1 | Spotted fever due to *Rickettsia conorii* | Spotted fever rickettsioses |
|  | A77.2 | Spotted fever due to *Rickettsia siberica* | Spotted fever rickettsioses |
|  | A77.3 | Spotted fever due to *Rickettsia australis* | Spotted fever rickettsioses |
|  | A77.8 | Other spotted fevers | Spotted fever rickettsioses |
|  | A77.9 | Spotted fever, unspecified | Spotted fever rickettsioses |
|  | A77.4 | Ehrlichiosis | Ehrlichiosis |
|  | A77.40 | Ehrlichiosis, unspecified | Ehrlichiosis |
|  | A77.41 | Ehrlichiosis, *E. chaffeensis* | Ehrlichiosis |
|  | A77.49 | Other ehrlichiosis | Ehrlichiosis |
|  | A79 | Other rickettsioses | Other rickettsial disease |
|  | A79.0 | Trench fever | Other rickettsial disease |
|  | A79.1 | Rickettsialpox due to *Rickettsia akari* | Other rickettsial disease |
|  | A79.89 | Other specified rickettsioses | Other rickettsial disease |
|  | A79.9 | Rickettsiosis, unspecified | Other rickettsial disease |
|  | A79.81 | Rickettsiosis due to *Ehrlichia sennetsu* | Other rickettsial disease |
