# Supplementary material for: Patient characteristics, treatment patterns, and outcomes of Rickettsial diseases among a commercially insured population in the United States, 2005–2017
Source: Sci Rep. 2021 Sep 15;11:18382. doi: 10.1038/s41598-021-96463-9 (PMC8443668; doi:10.1038/s41598-021-96463-9)
Supplement: Supplementary file 2 — Supplementary Information 2. [file 41598_2021_96463_MOESM2_ESM.docx]

**Supplemental Figure 1.**

**A. Summary of interval from index diagnosis to doxycycline prescription (in days), <8 years of age**

**
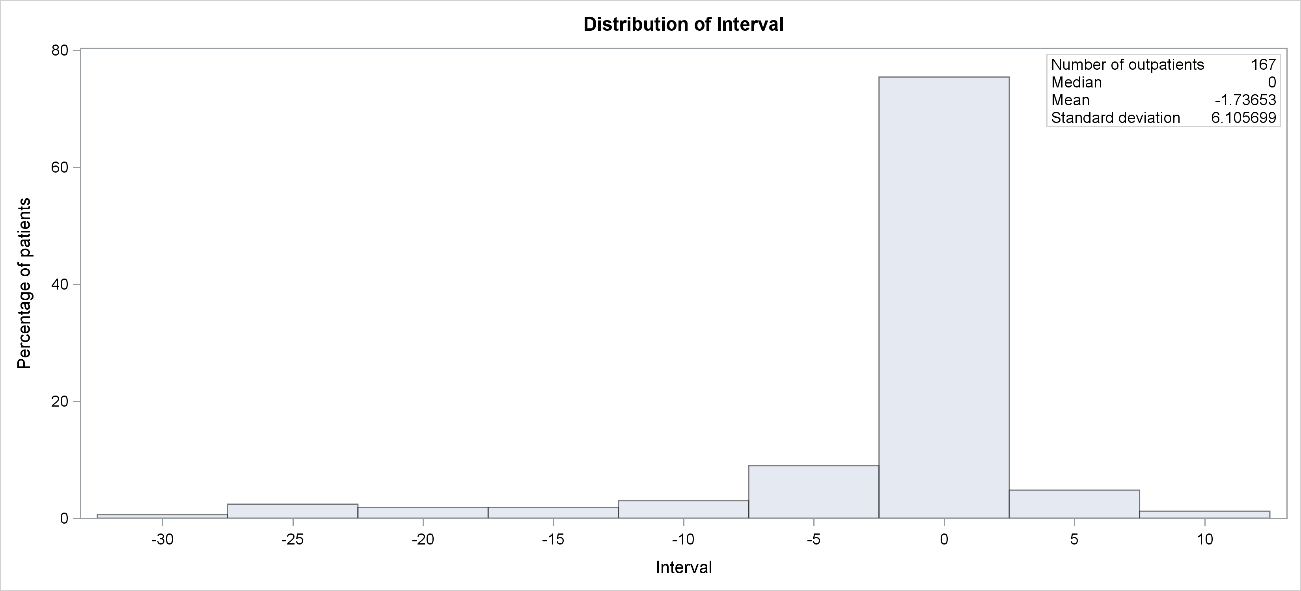
**

**B. Summary of interval from index diagnosis to doxycycline prescription (in days), 8 to <18 years of age**

**
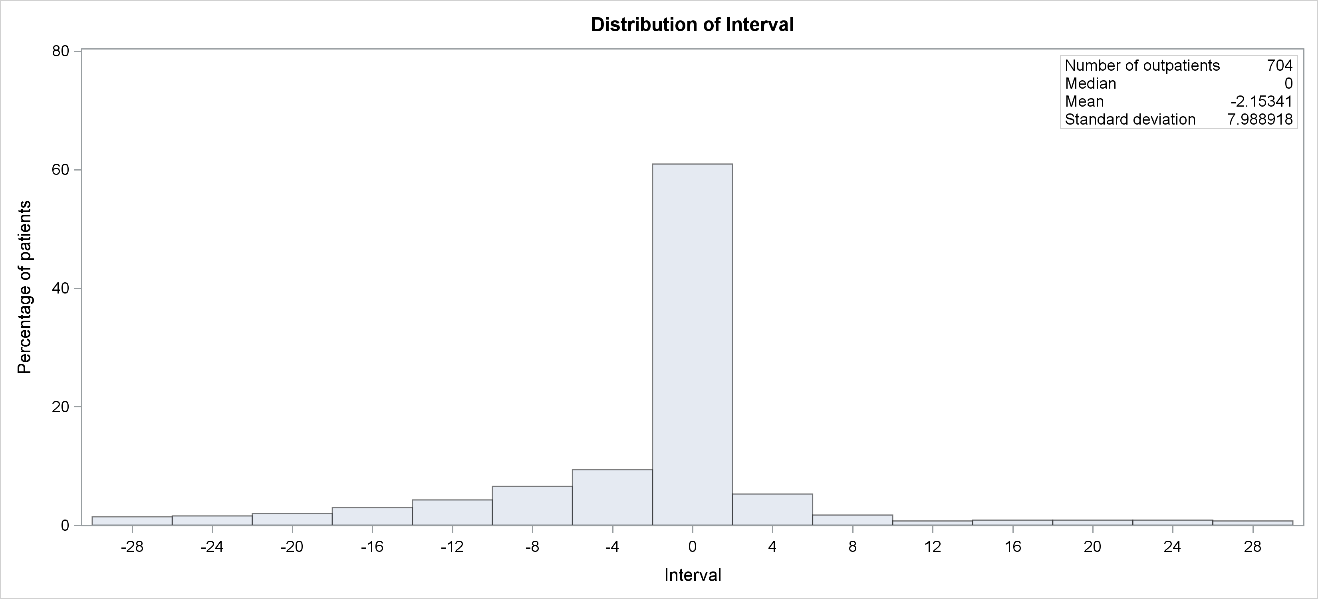
**


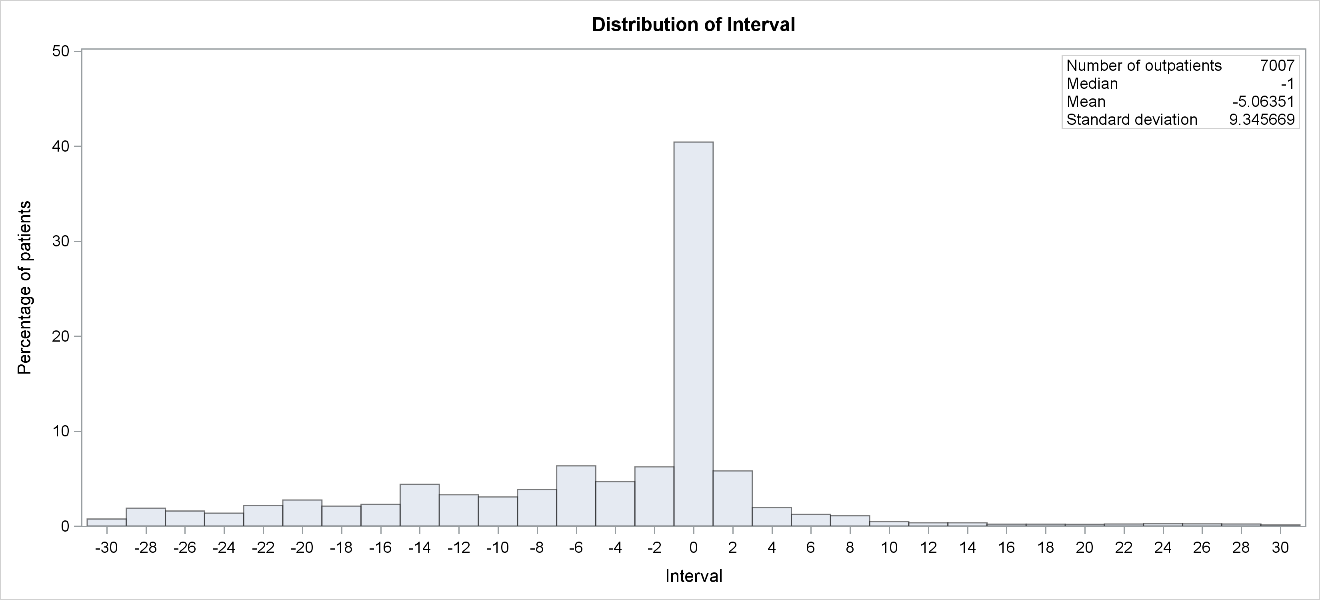
**C. Summary of interval from index diagnosis to doxycycline prescription (in days), 18 years of age and older**
